# Practical Notes and Formulæ

**Published:** 1885-01-20

**Authors:** 


### PRACTICAL NOTES AND FORMULÆ.

**Cough Mixture.**—

℞. Syrup of tar, ........ }
Syrup of ipecac ..... } aa .......................... ℥ ij.
Syrup of wild cherry }

M. S.: Teaspoonful four times a day.

**Delirium Tremens.**—

℞, Chloral hydrat ...................................... ʒ ij

Gum camphor ....................................... ʒ iss

Morph. sulphate .................................... gr. iv.

Olive oil, ad ........................................ ℥ iv.

M. Sig.: ʒ ij per enema every four to six hours.—*Naphey.*

**For Flatulence and Weak Stomach.**—

℞. Tinct. nux vomicæ ................................ gtt. v.

Tinct. gentian comp .... }

Tinct. [redacted] comp. . } aa ................... ʒ j.

Taken at one dose, before meals.

**Chronic Diarrhœa.**—

℞. Pulv. opium ...................................... gr. vj.
Ext. nux vomicæ ................................... gr. iij.
Cupri sulphas ..................................... gr. j.

Divide into twelve pills; one three times a day.—*Naphey.*

**Chronic Coughs.**—

℞. Muriate ammonia .................................. ℥ ss.

Fl. ext. wild cherry }

Syrup of tar ...... } aa ........................... ℥ ij.

Muriate morphia ................................... gr. j.

M. One teaspoonful every six hours.

**For Asthma.**—

℞. Tinct. assafœtida ................................ ℥ ss.

Syrup of tar, . . }

Tinct. lobelia,... } aa ............................ ℥ j.

M. Sig.: Take one to two teaspoonsful every two to four hours.

**For Mennorrhagia.**—

℞. Sulphate cinchoniæ ............................... ʒ ij.

Water ............................................. ℥ iij.

Elix. vitriol ...................................... ʒ ij.

Fl. ext. ergot .................................... ʒ iij.

M. Teaspoonful every three hours.

**Strychnia and Iron in Erysipelas.**—A writer in the American Medical Journal says, of his experience in epidemic erysipelas:

I thought of strychnia; and, combining it with tinct. ferri mur., it operated like magic. In a few hours my first patient was greatly improved.

℞. Tinct. ferri mur ............................. gtt. xx.
King's solution strychnia ......................... gtt. v.
Mix. To be given every two hours in a little water.

I thought this improvement might only be accidental, but an experience of twenty years has convinced me that strychnia and iron combined are the remedies in erysipelas.

**For Colic.**—

℞. Chloroform ............... } aa ........... ½ drachm.

Aromatic spirits of ammonia, }

Mint water ................................ ½ ounce.

To be given at a dose.

**For Hysteria.**—

℞. Tinct. assafœtida ......................... 2 drachms.
Carbonate of ammonia ....................... 20 grains.
Camphor water, to make ...................... 4 ounces.
M. Dose, a tablespoonful occasionally.

**For Whooping Cough.**—

℞. Aromatic spirits ammonia ................... ʒ j.
Spirits of ether ............................ ʒ j.
Tinct. of belladonna ........................ drops x.
Syrup of wild cherry ........................ 2 ounces.
Mix. Teaspoonful in equal part of water, to a child two years old, every three to four hours.

**For Sore Throat.**—

℞. Chlorate potass ............................ 1 drachm.
Water ...................................... 8 ounces.
Tinct. belladonna ........................... 10 drops.
Mix. Teaspoonful every two hours.

**For Cough of Infants of Six Months Old, and under.**—

℞. Syrup of onions ............................ 1 ounce.
Syrup of tar ................................ 1 drachm.
Camph. water ............................... 2 ounces.
Mix. Half to one teaspoonful every two to three hours.

**Expectorant.**—

℞. Fl. ext. wahoo ............................ ʒ vi.

Bicarb. potass ............................. ʒ iiss.

Morphia sulph .............................. grs. iij.

Fl. ext. ipecac ............................ gtts. xl to lx.

Fl. ext. squill ............................ ʒ iss.

Syr. tolu, to make ......................... ℥ vi. M.

Teaspoonful every two or three hours.—*Summary.*

**Bromides in Nausea and Vomiting.**—Dr. Cheron (Archives de Toxocologie) has found that great benefit results from the administration of bromides in an effervescing mixture in the persistent nausea and vomiting so often seen in women with uterine affections. His formula is :

No. 1.

℞. Bi-carb. potass..............................grs. xxx.
Aqua..............................fl. oz. ij.
Bromide potass..............................grs. xxx.
M.

No. 2.

℞. Citric acid..............................ʒ j.

Water..............................fl. ℥ iv.

Syrup..............................fl. ʒ x.

M.

Sig. : Add a teaspoonful of No. 1, to a teaspoonful of No. 2, and drink immediately. This dose may be repeated every half hour or hour, the quantity stated in the above formula being the maximum per diem amount. In localized pelvic peritonitis, this mixture often arrests the tendency to vomit, even in the acute stage.—*Phil. Med. News.*

**For Chronic or Persistent Ague.**—

℞. Argenious acid..............................grs. ij,
Strychnia sulph..............................grs. ij,
Sulph. quinine..............................ʒ j,
Querenes iron..............................ʒ j,
Ext. gentian..............................grs. xx.
M. Make pills xl.

Sig. : One three times daily, after eating.

The same formula, with strychnia and arsenic in from 1-20 to 1-40 of a grain, as needed, has been used for many years past as the most reliable remedy in chorea. Same mode of administering.—*Ib.*

**Chronic Chills** —

Dr. Charles J. Fox, of Williamantic, Conn., says that, for several years past, I have used the following with remarkable success in the treatment of malarial affections of the most aggravated type :

℞. Chinodinæ..............................grs. ccxx

Acid sulph., q. s. ad. solv.

Ol. pip. nig..............................ʒ ss,

Ol. limonis..............................ʒ j,

Alcohol, q. s. ad. solv. M.

Et adde :

℞. Aqua, q. s. ad..............................O j,

Syrup..............................O j.

Sig. : One teaspoonful every four hours.—*Ib.*

**For Flatulence and Weak Stomach.**—

℞ Tinct. nux vomica........................ gtt. v

Tinct. gentian comp., } aa .................. ℥ j.

Tinct. cardamom comp., }

Taken at one dose, before meals.

